# Actin Cytoskeletal Dynamics in Single-Cell Wound Repair

**DOI:** 10.3390/ijms221910886

**Published:** 2021-10-08

**Authors:** Malene Laage Ebstrup, Catarina Dias, Anne Sofie Busk Heitmann, Stine Lauritzen Sønder, Jesper Nylandsted

**Affiliations:** 1 Membrane Integrity, Danish Cancer Society Research Center, Strandboulevarden 49, 2100 Copenhagen, Denmark; male@cancer.dk (M.L.E.); cad@cancer.dk (C.D.); anhe@cancer.dk (A.S.B.H.); stilau@cancer.dk (S.L.S.); 2 Department of Cellular and Molecular Medicine, Faculty of Health Sciences, University of Copenhagen, Blegdamsvej 3C, 2200 Copenhagen, Denmark

**Keywords:** actin dynamics, cell membrane, cortical actin cytoskeleton, mechanosensing, membrane resealing, membrane restructuring, plasma membrane injury, plasma membrane repair, single-cell wound repair, wound healing

## Abstract

The plasma membrane protects the eukaryotic cell from its surroundings and is essential for cell viability; thus, it is crucial that membrane disruptions are repaired quickly to prevent immediate dyshomeostasis and cell death. Accordingly, cells have developed efficient repair mechanisms to rapidly reseal ruptures and reestablish membrane integrity. The cortical actin cytoskeleton plays an instrumental role in both plasma membrane resealing and restructuring in response to damage. Actin directly aids membrane repair or indirectly assists auxiliary repair mechanisms. Studies investigating single-cell wound repair have often focused on the recruitment and activation of specialized repair machinery, despite the undeniable need for rapid and dynamic cortical actin modulation; thus, the role of the cortical actin cytoskeleton during wound repair has received limited attention. This review aims to provide a comprehensive overview of membrane repair mechanisms directly or indirectly involving cortical actin cytoskeletal remodeling.

## 1. Introduction

**Figure 1:**
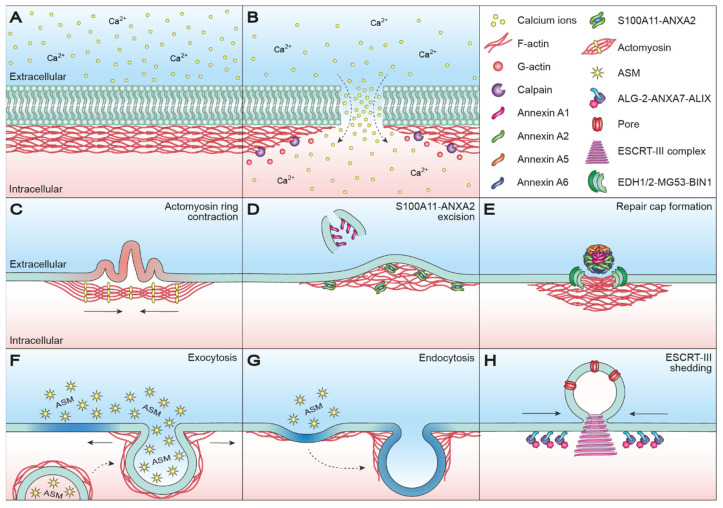
Actin-associated plasma membrane repair mechanisms in single-cell wound repair. (**A**) The PM and underlying cortical actin cytoskeletal are intact and maintaining cellular homeostasis. (**B**) Disruption of the PM causes a rapid influx of Ca^2+^, which causes depolymerization of actin filaments to G-actin monomers primarily through Ca^2+^-dependent activation of intracellular calpain proteases. Moreover, the increase in intracellular Ca^2+^ activates and recruits repair machinery for membrane resealing. (**C**) Subsequent to membrane patching, PM restoration in *Xenopus* oocytes occurs by contraction of an actomyosin ring anchored to the uninjured membrane at regular intervals. Ring contraction pushes injured membrane (red) outward, while dragging uninjured membrane (green) inward. (**D**) S100A11-ANXA2-dependent actin polymerization promotes wound closure by pulling the membrane edges towards each other for resealing and excision of injured membrane marked by ANXA1. (**E**) In muscle cells, formation of an annexin-rich repair cap, consisting of ANXA1, ANXA2, ANXA5 and ANXA6, and an underlying clearance zone of F-actin are required for efficient sarcolemma repair. An adjacent shoulder of repair proteins, EDH1/2, MG53 and BIN1, further aids the repair process. (**F**) Injury-induced lysosome-exocytosis reduces in-plane tension and generates ceramide microdomains (blue) through secretion of ASM. Mesoscopic modulation of the actin cytoskeleton is a prerequisite for vesicular exocytosis, since cortical actin comprises a mechanical barrier preventing vesicle fusion. At the same time, vesicle fusion cannot occur without some degree of actomyosin-mediated assistance. (**G**) ASM-generated ceramide microdomains (blue) trigger membrane invaginations, thereby priming the membrane for endocytosis-mediated restructuring. Because several steps of the endocytic process require delicate actin modulation, the internalization of injured membrane regions is highly dependent on actin dynamics. (**H**) ESCRT-III-mediated shedding of pore-injured membrane regions through spiral contraction. Ca^2+^-dependent formation of ALG-2-ANXA7-ALIX complexes at the injury site promotes the sequential recruitment of ESCRT-III components, resulting in complex assembly and microparticle shedding. ANXA: annexin; ALG-2: apoptosis-linked gene-2; ALIX: ALG-2-interacting protein X; ASM: acid sphingomyelinase; BIN1: Myc box-dependent-interacting protein 1; Ca^2+^: calcium ions; EDH1/2: EH-domain-containing protein 1/2; ESCRT-III: endosomal sorting complex required for transport III; G-actin: globular actin; MG53: Mitsugumin 53; PM: plasma membrane; S100A11: S100 calcium binding protein A11; Vps4B: vacuolar protein sorting-associated protein 4B.

The plasma membrane (PM) constitutes a physical barrier protecting cells against external forces, thereby maintaining cellular homeostasis and ensuring cell survival [1,2,3,4,5]. The eukaryotic PM consists of a 5–10 nm lipid bilayer with a diverse assortment of integral proteins, which translate external stimuli (e.g., secreted signaling molecules and tension induced by cell-cell adhesion) into intracellular signaling transduction and cellular responses [6]; thus, the PM not only shields cells against the extracellular milieu, but also permits cellular communication with the surrounding environment. Beneath the PM lies a meshwork of actin filaments, known as the cortical actin cytoskeleton, which strengthens and supports the PM through direct attachments (Figure 1A) [7]. In addition, actin filaments together with microtubules and intermediate filaments are collectively responsible for maintaining cell shape and organization [8]. In addition to being an instrumental structural component, the actin network plays a central role in both mechanical and biochemical signaling, whereby actin-dependent forces, amongst others, are essential for cell migration, intracellular trafficking, cytokinesis and mechanosensing [9]. This functional diversity originates from a vast array of actin regulatory proteins [10,11,12], which together with a large pool of globular (G)-actin monomers distributed throughout the cell allow rapid and dynamic modulation of the actin cytoskeleton without the need for prior transcriptional regulation [13]. These characteristics make the cortical actin cytoskeleton highly suitable for a role in PM repair; however, despite the many known functions of actin, the mechanisms underlying actin cytoskeletal remodeling remain rather elusive.

Throughout the lifetime of a cell, membrane integrity can become compromised under different physiological and pathological conditions, such as skeletal muscle contraction and pore formation by toxins [2,3,14]. Loss of membrane integrity ultimately threatens cell survival, although the resultant loss of homeostasis can also drive acute and chronic pathologies (e.g., by causing calcium toxicity and chronic inflammation leading to tissue degeneration) [3]. For this reason, cells are equipped with efficient repair mechanisms to reestablish membrane integrity. Despite subtle differences across cell and injury types, the repair mechanisms are evolutionarily conserved, highlighting the importance of membrane integrity for cell survival. Both insufficient and excessive repair capacities are associated with different pathologies, such as myopathies and cancer. An example is Duchenne muscular dystrophy, in which mutations in the gene encoding dystrophin, linking cytoskeletal filamentous (F)-actin with the extracellular matrix, make muscle cells more susceptible to mechanical damage [15]; therefore, mechanistic insights into membrane repair could potentially lead to novel therapeutic strategies [1,16,17].

Studies investigating membrane repair have often focused on membrane fusion events (via exocytosis-mediated repair), removal of injured membranes (by endocytosis-mediated repair or shedding) and recruitment or assembly of protein repair complexes (e.g., annexins and endosomal sorting complex required for transport (ESCRT)-III) [1,14,18,19,20]; however, the implication of the cortical actin cytoskeleton in membrane resealing and restructuring has received limited attention, despite the undeniable need for rapid and dynamic cytoskeletal changes in response to membrane damage and repair. Moreover, cellular adaptions to mechanical stimuli through actin-based mechanosensing must be tightly linked with membrane repair to orchestrate an appropriate and efficient repair response following mechanical damage. This review aims to provide a comprehensive overview of membrane repair mechanisms directly or indirectly involving cortical actin cytoskeletal remodeling and further touches upon the role of actin-based mechanosensing.

## 2. Cortical Actin Dynamics in Membrane Resealing

Disruption of the PM causes a rapid influx of calcium ions (Ca^2+^) from the extracellular milieu due to a 10,000-fold concentration gradient across the PM (Figure 1B) [21]. Accordingly, the wound size is proportional to the level of Ca^2+^ influx, which in turn activates and recruits repair machinery for membrane resealing [14,22]; therefore, both the source or type of damage and the wound size are determining factors for the initial and follow-up responses.

Importantly, Ca^2+^ influx not only activates and recruits repair machinery, but also drives actin cytoskeletal remodeling by depolymerizing actin filaments to G-actin [23,24,25,26]. Activation of intracellular calpain proteases is likely the primary cause of injury-induced cytoskeletal remodeling, as calpains have been demonstrated to be required for Ca^2+^-dependent single-cell wound repair by cleaving downstream targets such as the intermediate filament, vimentin, and the actin–integrin linker, talin [25,26]. Disassembly of the cortical actin cytoskeleton is necessary, as the tensile forces generated by the cytoskeleton, which are essential for normal cell function and morphology, prevent efficient membrane repair. For smaller wounds, actin cytoskeleton relaxation may be enough to drive thermodynamically favored membrane resealing [4,27,28]; however, larger wounds require the assistance of a specialized repair machinery, which also depends on preliminary tension relief through Ca^2+^-mediated cortical actin depolymerization. In fact, compound-induced F-actin stabilization inhibits membrane resealing, whereas compound-induced F-actin depolymerization has been shown to enhance single-cell wound repair [29]. Actin re-polymerization succeeds the initial Ca^2+^-dependent depolymerization and cooperates with specialized repair machinery to mediate PM repair through a variety of mechanisms. Besides directly aiding PM resealing, the cortical actin cytoskeleton needs to undergo rapid and dynamic changes in order to meet the requirements of efficient membrane repair, essentially making all PM repair mechanisms dependent on actin cytoskeletal dynamics. 

### 2.1. Wound Healing in Oocytes

Oocytes of *Xenopus laevis* and *Echinoidea* have often been used to study single-cell wound repair, as their large size and lack of tension induced by cell–cell adhesion provide a simpler model to study repair compared to somatic cells. Oocytes have been reported to recover from large mechanical disruptions of the PM and underlying cytoskeleton (>1000 um^2^) [30,31,32]. The rapid Ca^2+^ influx accompanying wounding stimulates immediate homotypic fusion of yolk granules at the injury site, resulting in the formation of a large ‘membrane patch’ that fuses with the wound edges in a Ca^2+^-dependent manner to reseal the damaged membrane [30,31,32]. Yolk granules serve as a readily available membrane source for patching in oocytes due to their abundance and close proximity to the PM. Initially, lysosomes and other non-secretory vesicles and organelles (e.g., enlargeosomes and mitochondria) were proposed to take part in a type of patch-like formation at the injury site in somatic cells [33,34,35,36]; however, these vesicles and organelles would in all likelihood not fulfill the spatiotemporal requirements for rapid and efficient membrane repair (reviewed in [3,37,38,39,40,41]). Interestingly, a recent study demonstrated a type of patch-mediated membrane repair in *Dictystelium* cells, in which de novo synthesis of vesicles at the injury served as the membrane source for patching [42]; to date, this phenomenon has only been demonstrated in *Dictystelium* cells, which is why patch-mediated repair is generally more controversial in somatic cells.

**Figure 2:**
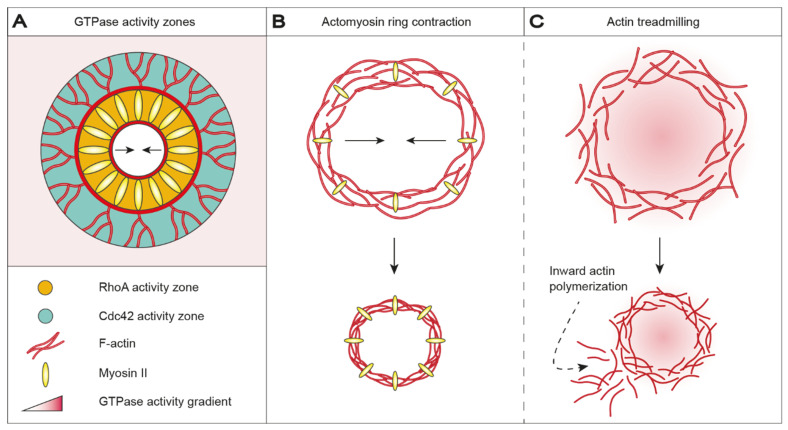
Wound healing in *Xenopus* oocytes is accomplished by contraction of an actomyosin ring or actin treadmilling. (**A**) Actomyosin ring positioning, assembly and contraction are orchestrated by demarcated GTPase activity zones. Injury-triggered Ca^2+^ influx recruits and activates RhoA (orange) and Cdc42 (green), which accumulates in mutually exclusive, concentric activity zones around the wound. RhoA and Cdc42 co-localize with myosin II and actin, respectively. RhoA stimulates polymerization of stable, unbranched actin filaments in the inner zone, whereas Cdc42 stimulates polymerization of dynamic, branched actin filaments in the outer zone. (**B**) In detail, RhoA controls actomyosin contractibility by influencing the activity of MLCK and ROCK, which in turn regulates myosin II light chain phosphorylation levels. Accordingly, wound closure is promoted by actomyosin-based ring contraction. (**C**) Alternatively, contraction-independent wound closure can be mediated through actin treadmilling. Here, a directional GTPase activity gradient from the leading edge to the trailing edge of the wound results in inward actin polymerization, which constricts and eventually seals the wound; however, actin treadmilling results in less organized and discontinuous wound closure. Ca^2+^: calcium ions; Cdc42: cell division control protein 42 homolog; MLCK: myosin light-chain kinase; RhoA: Ras homolog family member A; ROCK: Rho-associated kinase.

In *Xenopus* oocytes, *Echinoidea* oocytes and *Drosophila* early embryos, membrane integrity is reestablished by the formation and contraction of an actomyosin purse string structure [43,44,45,46,47]. An actomyosin ring is anchored to the uninjured membrane at regular intervals and aids PM restoration by pushing the injured membrane outward while dragging the uninjured membrane inward as the ring contracts (Figure 1C) [45]. Actomyosin ring positioning, assembly and contraction are broadly conserved but vary with respect to GTPase orchestration and microtubule assembly and function [48,49]. In *Xenopus* oocytes, injury-triggered Ca^2+^ influx recruits and activates Ras homolog family member A (RhoA) and cell division control protein 42 homolog (Cdc42), which localize in mutually exclusive, concentric zones around the wound (Figure 2A) [44,48]. RhoA co-localizes with myosin II at the leading edge of the wound and is encircled by an outer ring of Cdc42 co-localizing with actin [48,50]. RhoA is believed to promote polymerization of stable, unbranched actin filaments at the wound edge through the recruitment and activation of actin nucleator diaphanous (Dia) and protein kinase N (Pkn) [51]. Cdc42 promotes the polymerization of dynamic, branched actin filaments at the wound periphery via activation of neural Wiskott–Aldrich syndrome protein (N-WASP), which in turn activates actin-related proteins-2/3 (Arp2/3) [52], consequently providing direct evidence that membrane repair requires both depolymerization and subsequent re-polymerization of actin. A microtubule network accumulates radially around the wound and recruits Arp2/3 and myosin II, thereby assisting in organizing ring assembly and contraction [43,53]. RhoA indirectly regulates myosin II light-chain phosphorylation by influencing the activity of myosin light-chain kinase (MLCK) and Rho-associated kinase (ROCK) [54,55], thereby mediating microtubule-dependent ring contraction (Figure 2B). Remarkably, pharmacological inactivation of myosin failed to prevent wound closure in *Xenopus* oocytes, demonstrating that myosin-mediated contraction is dispensable for wound closure [50]; however, the ring structure became less organized and wound closure occurred discontinuously upon myosin inactivation. Contraction-independent wound closure is mediated through a rapid turnover of actin, termed actin treadmilling, arising from a directional gradient in GTPase activity (Figure 2C). Decreasing GTPase activity from the leading edge to the trailing edge of the wound promotes inward actin polymerization, which constricts and eventually seals the wound. Actin treadmilling is likely outplayed or complemented by myosin-mediated contraction during undisturbed wound closure, for which reason our understanding of actin treadmilling in membrane and cytoskeletal restructuring is limited. 

Actomyosin ring assembly and contraction are tightly controlled by GTPase orchestration, as GTPase activity modulates actin and myosin dynamics. GTPase patterning has been proposed to form in response to the injury-induced reorganization of lipid domains at and proximal to the injury site [56]. Wounding of *Xenopus* oocytes triggers the formation of domains specifically enriched with phosphatidylinositol 4,5-diphosphate (PIP_2_), phosphatidylinositol 3,4,5-trisphosphate (PIP_3_), phosphatidylserine (PS), phosphatidic acid (PA) and diacylglycerol (DAG). PS localizes at the zone of high RhoA activity closest to the wound, whereas PIP_2_ and PIP_3_ localize to the outer ring of high Cdc42 activity. Both PA and DAG localize to an intermediate zone with overlapping RhoA and Cdc42 activity. DAG-enriched domains are able to recruit and activate protein kinase C (PKC) β and η, which may act as upstream regulators by stimulating and inhibiting RhoA and Cdc42 activity, respectively [56]; thus, injury-induced changes in PM composition may indirectly aid GTPase patterning, which in turn promotes PM restoration through actomyosin ring assembly and contraction.

Actomyosin structures are evolutionarily conserved and are involved in different cellular processes in somatic cells (e.g., in cytokinesis and cell extrusion) [57]; however, they have not been implicated in PM repair. Somatic cells appear instead to be dependent on other types of highly specialized repair mechanisms, despite possessing the necessary machinery to form a contractile ring structure; however, the reason why somatic cells refrain from using such structures in single-cell wound repair is currently unknown [58]. Mechanisms based on other proteins may outcompete contractile ring structures in a repair context. For instance, annexins are capable of inducing membrane curvature and constriction during PM repair and are further capable of modulating actin dynamics [59,60].

### 2.2. Would Healing in Somatic Cells

#### 2.2.1. Protein-Mediated Plasma Membrane Repair

Multiple members of the highly conserved Ca^2+^-binding annexin protein family are enriched at the injury site and play a direct or indirect role in the regulation of actin re-polymerization [1,4,60,61]. Annexin A2 (ANXA2) and one of its interacting partners, S100 calcium binding protein A11 (S100A11), alter local actin dynamics in a direct manner—S100A11 and ANXA2 bind to F-actin and restrict the depolymerization of existing actin fibers (Figure 1D) [62,63]. Actin re-polymerization may also be aided by the self-assembly of another annexin family member, ANXA5, into 2D arrays around the wound edge, which is responsible for generating contractive forces [60,64]. Ca^2+^-mediated formation of S100A11-ANXA2 complexes has been described to augment PM repair in invasive MCF7 breast cancer cells expressing a truncated version of the ErbB2 receptor (MCF7-p95ErbB2) [60]. The cumulative effect of annexin-mediated modulation of the actin cytoskeleton is evident following wounding of MCF7-p95ErbB2 cells. In this case, Ca^2+^ influx triggers rapid local actin depolymerization and the recruitment of ANXA1 to the injury site, followed by Ca^2+^-dependent S100A11-ANXA2 complex formation and its translocation to membranes adjacent to the injury. Here, S100A11-ANXA2 complexes stimulate actin polymerization, which functions as a local repair zone, since buildup of F-actin promotes wound closure by pulling the membrane edges towards each other for eventual resealing and excision of damaged membrane marked by ANXA1 (Figure 1D) [60]. S100A11 and ANXA2 are overexpressed in various types of cancer and their overexpression is associated with tumor metastasis and poor prognosis [65,66,67,68,69,70,71,72,73,74,75,76,77]. In fact, loss of S100A11-ANXA2 complexes impairs PM repair and the invasiveness of MCF7-p95ErbB2 cells [60]. This highlights the therapeutic potential of targeting repair mechanisms in pathologies, such as cancer, in which cells are highly dependent on efficient PM repair for survival.

Neuroblast-differentiation-associated protein AHNAK (AHNAK) is known to form a complex with S100A11-ANXA2 (2:2) in a Ca^2+^-dependent manner [78,79,80]. The AHNAK-S100A11-ANXA2 heterotetramer has been proposed to regulate cortical actin cytoskeletal organization and the membrane cytoarchitecture through the ability of AHNAK to interact with both G- and F-actin [81,82], suggesting that AHNAK-S100A11-ANXA2 regulates the membrane cytoarchitecture by directly interacting with the cortical actin cytoskeleton. Accordingly, AHNAK depletion hinders cortical actin cytoskeleton reorganization [82]. Moreover, AHNAK is associated with enlargeosomes, which have been shown to undergo exocytosis in response to injury-induced Ca^2+^ influx (see Section 2.2.2) [83,84,85]; however, the role and mechanism of enlargeosome exocytosis in PM repair is still not well defined, presumably due to lack of focus in favor of other repair mechanisms.

In contrast to cancer cells that often display increased repair capacity, muscle cells often display deficient repair capacity translating into different pathologies. Swaggart et al. identified ANXA6 as a crucial modifier of muscular dystrophy [86]. Upon membrane injury, ANXA6 orchestrates into a clearly demarcated zone, termed a repair cap, at the injury site and mediates membrane repair (Figure 1E). A carboxy terminally truncated version ANXA6, incapable of membrane binding due to the lack of its PS-binding domain, was shown to disrupt repair cap formation and result in sarcolemmal leakiness [86]. Repair cap formation was later shown to be both Ca^2+^- and actin-dependent [87]. F-actin exhibits a temporal recruitment pattern identical to ANXA6, accumulating within 14 seconds post-injury [87]. Interestingly, ANXA6 and F-actin accumulate in mutually exclusive zones with F-actin accumulating in a so-called clearance zone beneath the repair cap. Compound-induced inhibition of F-actin formation significantly delayed repair cap formation, whereas the removal of exogenous Ca^2+^ completely abolished repair cap formation, demonstrating that Ca^2+^ and actin re-polymerization are needed for ANXA6 translocation and repair cap formation, presumably facilitating sarcolemma repair by creating a cytoplasmic located plug that reduces the exchange with the extracellular milieu. In addition to ANXA6, annexin family members ANXA1, ANXA2 and ANXA5 are recruited to the injury site and partake in repair cap formation (Figure 1E) [87]. The cumulative effect of this multimeric annexin complex on actin dynamics and vice versa is poorly understood, yet the annexin-rich repair cap and underlying F-actin structure must work together in a cooperative fashion, since both parties are required for efficient sarcolemma repair. An adjacent shoulder of repair proteins, including dysferlin, EH domain-containing protein 1 (EHD1), EDH2, Mitsugumin 53 (MG53) and Myc box-dependent-interacting protein 1 (BIN1), further aids the repair process, and all proteins are required for optimal sarcolemma repair [87]. 

There is a delicate balance in membrane repair efficiency, as both insufficient repair, as observed in myopathies, and excessive repair, as observed in cancer, comprise major pathological triggers; therefore, increased insights into the mechanistic facets of PM repair, including actin dynamics, would open up largely unexplored therapeutics avenues applicable for a wide range of pathologies.

#### 2.2.2. Exocytosis- and Endocytosis-Mediated Plasma Membrane Repair

In exocytosis-mediated repair, different intracellular compartments and organelles (including endosomes, enlargeosomes, reserve or secretory granules and lysosomes) fuse with the PM at the injury site (Figure 1F) [30,32,33,37,88,89,90,91,92,93,94,95,96]. Membrane patch formation comprises one model of exocytosis-mediated repair [30,31,32]. Andrews et al. proposed an alternative model in which exocytosis of intracellular membrane to the PM reduces in-plane tension, thereby bringing the wound edges closer together to promote membrane resealing facilitated by auxiliary repair mechanisms (Figure 1F) [1,60,97,98,99]. Accordingly, exocytosis-mediated reduction of in-plane tension has been demonstrated to be crucial for the repair of various types of membrane injuries (reviewed in [3,100]). In somatic cells, lysosomes are proposed to be the main source of membrane material for exocytosis-mediated repair [33,96]; however, it is uncertain whether lysosome exocytosis is able to fulfill the spatiotemporal requirements for rapid PM repair, especially for larger wounds, since the majority of lysosomes are located in the perinuclear region and require transport to the injury site [37,39,40,41]. Microtubule-dependent trafficking facilitated by the motor proteins kinesin and dynein comprises the primary mechanism for long-distance vesicle transport [101,102]; however, microtubule-independent long-distance vesicle transport is possible and can be accomplished through actin nucleation [41]. Once close to the cell periphery, the cortical actomyosin network plays a central role in mediating vesicle anchoring, docking and fusion with the PM (reviewed in [103,104]); therefore, mesoscopic modulation of the cortical actomyosin network is needed to successfully execute exocytosis. On one hand the network comprises a mechanical barrier preventing vesicle fusion [29], while on the other hand vesicle fusion cannot occur without some degree of actomyosin-mediated assistance [103,104]. Supportive of this biphasic relationship, compound-induced F-actin depolymerization reduced the initial rate of exocytosis in mast cells by impairing actin-mediated vesicle capture and transport, but increased the total level of vesicle secretion by weakening the cortical actin barrier [105].

Long-distance trafficking of lysosomes from the perinuclear region to the cell periphery is expected to be too slow to fulfill the sub-second demand for patch-mediated repair [40,41]. Instead, exocytosis-mediated PM repair may primarily aim to reduce in-plane tension at areas flanking the injury site, thereby promoting repair through membrane relaxation. Furthermore, Andrews et al. proposed that exocytosis-mediated fusion of intracellular membrane not only serves to reduce in-plane tension, but also comprise a prerequisite for ensuing endocytosis-mediated repair and membrane restructuring [92]. Injury-induced lysosome exocytosis causes lysosomal secretion of acid sphingomyelinase (ASM) at the outer membrane leaflet, which cleaves the phosphorylcholine head of sphingomyelin to generate ceramide (Figure 1F) [106,107]. Formation of ASM-generated ceramide microdomains triggers membrane invaginations, thereby priming the damaged membrane regions for rapid internalization through both clathrin-dependent and -independent endocytosis (Figure 1G) [108,109,110]. Actin dynamics are instrumental in aiding in vesicle internalization, as actin supports several endocytic steps, including membrane invagination, scission of newly formed vesicles from the PM and vesicle trafficking away from the PM (reviewed in [111,112,113]). 

Taken together, exocytosis- and endocytosis-mediated repair appear to comprise a tandem process in which initial in-plane tension release promotes membrane resealing, followed by ASM driven membrane restructuring to reestablish membrane integrity. Moreover, endocytosis has long been considered the primary repair mechanism for pore-mediated membrane injuries arising under both physiological and pathological conditions (e.g., cytolysins secreted by immune cells and bacteria) [37,88,109,114]. Here, pore-injured membrane regions are internalized and targeted for lysosomal degradation; however, a recent study questioned the direct implication of endocytosis in pore removal and instead suggested that endocytic mechanisms regulate membrane composition and in-plane tension in response to pore-mediated injuries [115]. Regardless of the exact role of exocytosis- and endocytosis-mediated repair, the underlying mechanisms share the requirement for delicate modulation of actin cytoskeletal dynamics in order to aid efficient vesicle fusion and fission during single-cell wound repair. 

#### 2.2.3. Blebbing-Mediated Plasma Membrane Repair 

The formation of PM blebs (cytoplasmic spherical protrusions only connected to the cell body through a thin neck) resembles a reversed and unfinished form of endocytosis. Blebbing arises from disrupted PM–cytoskeleton interactions, which permit actomyosin contraction independent of the membrane, which instead becomes inflated through intracellular osmotic pressure [116,117]. There is some discrepancy regarding the underlying mechanism(s) of blebbing, as some studies have reported bleb formation to be driven by Ca^2+^-dependent actin depolymerization [37,118], while others have argued that it is a Ca^2+^-independent passive process, resulting from the formation of spatially organized lipid domains in response to toxin oligomerization [119,120]. Likewise, the purpose of blebbing has been debated, arising to compartmentalize pores (protecting the cell from Ca^2+^ toxicity and loss of cytosolic content) as a prerequisite for membrane restructuring through microparticle shedding or for cortical actin regeneration (see Section 3.2) [37,117,118,121,122,123]. Mechanistically, annexins, especially ANXA1, are key proteins in bleb formation because of their Ca^2+^ sensitivity, PS-binding domain and oligomerization properties, through which they can mediate membrane fusion [1,124]. Supportive of such a role, mass spectrometry analysis confirmed the presence of annexins inside shed blebs [125]. Alternatively, local actin reassembly within blebs can drive myosin II-dependent bleb retraction through a RhoA-ROCK-Rho family GTPase 3 (RND3) feedback loop [117,126,127,128], suggesting that blebbing could be involved in cortical actin cytoskeletal remodeling following PM injury. 

Regardless of discrepancies related to its underlying mechanism and role, the involvement of blebbing in PM repair is undeniable. A better understanding of actin dynamics during blebbing could provide a mechanistic and functional understanding of blebbing in PM repair and cytoskeletal restructuring. At the same time, studying the different stages of blebbing could perhaps provide insights into the sequence of events contributing to cortical actin cytoskeleton de- and reassembly.

## 3. Cortical Actin Dynamics in Membrane Restructuring

PM repair needs to occur within a sub-second window to ensure cell survival; hence, it comes at a cost, namely that it is crude and often unable to sustain complete PM function. As such, the resealed membrane and underlying cytoskeleton need to undergo extensive restructuring to reestablish membrane integrity [2,18,20,92]. The instrumental role of actin cytoskeletal dynamics in driving post-injury PM restructuring has received increasing attention in recent years and has resulted in novel insights into actin-driven membrane restructuring.

### 3.1. Macropinocytosis-Mediated Membrane Restructuring

**Figure 3:**
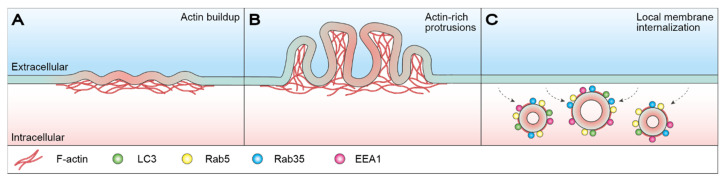
LC3-asscoiated macropinocytosis-mediated membrane restructuring. (**A**) Subsequent to membrane resealing, actin buildup occurs around the injury site (red) resulting in the formation of actin-rich membrane protrusions. (**B**) Actin-driven extension of PM ruffles generates cup-like structures that close to form large macropinosomes. (**C**) Internalization of macropinosomes via macroendocytic events helps restore membrane integrity as injured membrane regions are removed from the PM. Internalized macropinosomes are initially positive for actin, Rab5, Rab35, EEA1 and occasionally LC3, and eventually undergo potential size-dependent shrinkage before fusing with lysosomes for degradation and recycling. For more details see [129]. EEA1: early endosome antigen 1; LC3: light-chain 3; PM: plasma membrane; Rab5/35: Ras-related protein Rab-5/35.

We recently revealed that MCF7 breast carcinoma cells undergo a form of injury-triggered macrointernalization, termed light-chain 3 (LC3)-associated macropinocytosis, in which cells restructure their PM by internalizing large portions of damaged membrane [129]. Subsequent to membrane resealing, buildup of actin around the injury site results in the formation of actin-rich membrane protrusions. Here, the actin-driven extension of PM ruffles generates cup-like structures, which close to form large macropinosomes. Internalized macropinosomes undergo potential size-dependent shrinkage before eventually fusing with lysosomes for degradation and recycling. This process was demonstrated to take place approx. 8–10 min after injury and initial membrane resealing and was dependent on autophagy-related 7 protein (ATG7), sequestosome-1 (SQSTM1/p62) and Run domain Beclin-1 interacting and cysteine-rich-containing protein (Rubicon) (Figure 3) [129]. Compound-induced inhibition of macropinocytosis sensitized cells toward mechanical-injury-induced cell death, thereby accentuating the importance of membrane restructuring, as membrane resealing alone is insufficient in reestablishing membrane integrity and ensuring post-injury survival. Moreover, the data highlight the instrumental role of actin cytoskeletal dynamics in driving membrane restructuring through LC3-associated macropinocytosis [129]. 

### 3.2. Plasma Membrane Repair Facilitated by Microparticle Shedding

Microparticle or ectosome shedding comprises an alternative PM repair mechanism for the removal of pore-injured membrane regions in response to damage. Shedding, also known as ectocytosis, is capable of repairing both small (<100 nm) and large (micron-scale) membrane injuries, via the ESCRT-III complex or annexin proteins [123,130,131,132,133].

ESCRT-III-mediated shedding is initiated by Ca^2+^ influx resulting from pore-related membrane permeabilization, which triggers the formation of apoptosis-linked gene-2 (ALG-2)-ANXA7 complexes that become tethered to the injured membrane through the PS-binding domain of ANXA7 [19]. Here, ALG-2-ANXA7 recruits ALG-2 interacting protein X (ALIX), which in turn promotes the sequential recruitment of ESCRT-III components driving complex assembly of a spiral-shaped structure capable of shedding the damaged membrane through spiral-mediated contraction (Figure 1H) [130,131]. F-actin-driven formation of filopodia-like protrusions were found to act as scaffolds for microparticle shedding in HeLa cervical cancer cells, a process that was dependent on myosin 1A and ECSRT-III component vacuolar protein sorting-associated protein 4B (Vps4B) [134]. Membrane protrusions occurred 10–15 min post-injury, suggesting that shedding does not partake in the acute repair phase, but instead facilitates subsequent membrane and cytoskeletal restructuring. Scaffold construction was mediated by the relocation of actin from neighboring regions to the injury site in a process that was dependent on F-actin nucleation by N-WASP and myosin 1A [134]. Local actin depolymerization at these filopodia-like protrusions may prime shedding-mediated restructuring by causing membrane deformation, thereby favoring vesicle formation of the unsupported membrane. Previous studies have demonstrated compound-induced actin modulation to induce shedding in different cell types [135,136,137].

Alternatively, membrane-binding proteins capable of sensing or inducing high membrane curvature, such as annexins, could perhaps in cooperation with local actin remodeling prime shedding-mediated membrane restructuring. Blebbing is the most likely priming mechanism. As such, Ca^2+^ influx can trigger the sequential recruitment and binding of ANXA1, ANXA2 and ANXA6, with the recruitment kinetics depending on the Ca^2+^ sensitivity of the individual annexins [123,132,133]. Annexin binding seals damaged membrane regions. Next, both the damaged membrane regions and the annexins are shed from the cell as microparticles [123,132,133]. Overall, annexin- and ESCRT-III-mediated microparticle shedding require the same form of cytoskeletal remodeling, as observed in exocytosis-mediated PM repair, since microparticle shedding is an exovesicular process. 

Interestingly, syntaxin-2 (SYX-2) interacts with epithelial fusion failure 1 (EFF-1) to facilitate PM repair downstream of ESCRT-III in *C. elegans* [138], suggesting that the SYX-2-EFF-1 repair machinery is likely involved in late-stage repair important for reestablishing membrane integrity. In agreement with such a role, the SYX-2-EFF-1 repair machinery was demonstrated to be required for successful membrane repair and animal survival, thereby potentially adding an additional layer to repair-mediated membrane restructuring. Early actin polymerization and Ca^2+^-regulated ESCRT-III signaling were shown to promote the sequential recruitment of SYX-2 and EFF-1 to the injury site, once again highlighting the instrumental role of cortical actin cytoskeletal dynamics in late-stage membrane repair and restructuring. 

## 4. Perinuclear Actin Dynamics

Mechanosensing describes the ability of a cell to sense mechanical stimuli from the extracellular milieu [139]. The cytoskeleton is the primary facilitator of mechanotransduction, as tension levels within the cells are mediated by the cytoskeletal system. Cytoskeletal tension force can activate molecular sensors, and in this way initiate intracellular signaling cascades, resulting in an orchestrated cellular response towards the imposed stimuli. Intriguingly, a novel actin structure has been shown to form in the perinuclear region in response to mechanical stimuli and biochemical signals related to mechanotransduction [140,141]. Force-induced perinuclear actin remodeling could be considered a cellular attempt to protect the nucleus against mechanical-induced ruptures. F-actin accumulation in the perinuclear region occurs rapidly (<1 min) in response to stimuli and was found to be dependent on Ca^2+^ influx, calcium regulator calmodulin and actin polymerization factor inverted formin-2 (IFN2) [140,141]. Interestingly, wound healing in *Dictyostelium* cells following laserporation was also shown to depend on Ca^2+^ influx, calmodulin and subsequent actin polymerization [42]. The shared repair toolkit raises the question as to whether perinuclear actin remodeling is not limited to mechanosensing, but may also be involved in nuclear envelope repair. ESCRT-III has been reported to facilitate nuclear envelope repair [142], further supporting a potential overlap between PM and nuclear envelope repair mechanisms. 

## 5. Conclusions

Actin was originally believed to be sufficient to mediate single-cell wound repair, but a growing body of evidence suggests that repair of larger wounds requires the assistance of specialized repair machinery. This previously redirected the focus from actin cytoskeletal dynamics in wound repair in favor of dedicated repair proteins and mechanisms; however, the function of actin as an evolutionarily conserved facilitator of membrane resealing and restructuring is undeniable and has been understudied in recent times. Especially when considering the growing appreciation for the role of membrane restructuring in cell health, mechanistic insight into cortical actin dynamics during membrane restructuring is lacking and is of therapeutic value. For the sake of clarity, this review has distinguished between immediate membrane resealing and restructuring, although in reality, they are cooperative phases of the same tandem repair process. This interconnectivity is exemplified by the occasional mechanistic overlap between membrane resealing and restructuring; for example, blebbing and shedding have been proposed to partake in both immediate repair, membrane restructuring and cortical actin regeneration, highlighting the need for both membrane and cytoskeletal restructuring. Taken together, the cortical actin cytoskeleton is not merely a passive bystander in single-cell wound repair, but an active and dynamic player given that essentially all PM repair mechanisms are directly or indirectly dependent on cortical actin cytoskeletal remodeling.
